# Impaired health-related quality of life in adolescents with allergy to staple foods

**DOI:** 10.1186/s13601-016-0128-5

**Published:** 2016-09-30

**Authors:** Jennifer Lisa Penner Protudjer, Sven-Arne Jansson, Roelinde Middelveld, Eva Östblom, Sven-Erik Dahlén, Marianne Heibert Arnlind, Ulf Bengtsson, Ingrid Kallström-Bengtsson, Birgitta Marklund, Georgios Rentzos, Ann-Charlotte Sundqvist, Johanna Åkerström, Staffan Ahlstedt

**Affiliations:** 1 The Centre for Allergy Research, Karolinska Institutet, P.O. Box 287, 17177 Stockholm, Sweden; 2 Institute of Environmental Medicine, Karolinska Institutet, Stockholm, Sweden; 3 Department of Public Health and Clinical Medicine, Umeå University, Umeå, Sweden; 4 Sachs’ Children and Youth Hospital, Södersjukhuset, Stockholm, Sweden; 5 Department of Clinical Research and Education, Södersjukhuset, Karolinska Institutet, Stockholm, Sweden; 6 Swedish Council on Health Technology Assessment, SBU, Stockholm, Sweden; 7 Department of Learning, Informatics, Management and Ethics, and Medical Management Centre, Karolinska Institutet, Stockholm, Sweden; 8 Allergy Unit, Sahlgrenska University Hospital, Gothenburg, Sweden; 9 The Swedish Asthma and Allergy Foundation, Stockholm, Sweden; 10 Department of Health and Caring Sciences, Linnaeus University, Kalmar, Sweden

**Keywords:** Adolescents, Food allergy, Health-related quality of life

## Abstract

**Background:**

Cow’s milk, hen’s egg and wheat are staple foods in a typical western diet. Despite the ubiquity of these foods, the impact of staple food allergy on health-related quality of life (HRQL) amongst adolescents is incompletely understood. The aims of this study were to make use of the Swedish version of EuroPrevall’s disease-specific food allergy quality of life questionnaire-teenager form (FAQLQ-TF) and to investigate the association between objectively-diagnosed staple food allergy and HRQL amongst adolescents.

**Methods:**

In this cross-sectional study, 58 adolescents aged 13–17 years [n = 40 (69 %) boys] with objectively-diagnosed allergy to the staple foods cow’s milk, hen’s egg and/or wheat and living in Stockholm, Sweden were included. Adolescents completed the FAQLQ-TF, which has a corresponding scale of 1 = best HRQL, and 7 = worst HRQL. Overall HRQL and domain-specific HRQL were established. Adolescents also reported symptoms, adrenaline auto injector (AAI) prescription and presence of other food allergies. A history of anaphylaxis was defined among those reporting difficulty breathing, inability to stand/collapse, and/or loss of consciousness. Clinically different HRQL was set at a mean difference of ≥0.5.

**Results:**

Overall mean HRQL was poorer than average [mean: 4.70/7.00 (95 % CI 4.30–5.01)]. The domain risk of accidental exposure was significantly associated with clinically better HRQL than the domain allergen avoidance and dietary restrictions (mean difference = 0.76; p < 0.001). Girls had clinically worse, but not statistically significantly different mean HRQL than boys (mean difference = 0.71; p < 0.07). HRQL tended to be worse amongst those with allergies to more than three foods or an AAI prescription. The number and types of symptoms, including a history of anaphylaxis were not associated with worse HRQL.

**Conclusions:**

As ascertained via a food allergy-specific questionnaire, adolescents with staple food allergy report poorer than average HRQL, specifically in relation to emerging independence and the need for support. Girls have clinically worse HRQL than boys. The number and type of previous symptoms and history of anaphylaxis were not associated with worse HRQL.

**Electronic supplementary material:**

The online version of this article (doi:10.1186/s13601-016-0128-5) contains supplementary material, which is available to authorized users.

## Background

Food allergy affects 2–8 % of adolescents [1, 2]. In this group, health-related quality of life (HRQL) or ‘the effects of an illness and its consequent therapy upon a patient, as perceived by the patient [3], may be impacted [4–6], particularly in relation to social well-being and independence [7]. Further, adolescents with food allergy report worse overall HRQL compared to matched non-food allergic controls [4, 5], or to adolescents with other chronic conditions [4, 8]. Notably, these studies involved the use of generic HRQL questionnaires [4, 5, 8], which may not identify the subtleties of food allergy or issues specific to the disease [9]. To address this limitation, EuroPrevall’s food allergy-specific HRQL questionnaires were specifically developed and validated to glean insights into food allergy that cannot be ascertained by generic questionnaires [9, 10]. The self-reported adolescent version of this questionnaire, the food allergy quality of life questionnaire-teenager form (FAQLQ-TF), presents a unique means by which to capture perceptions of HRQL of adolescents with food allergy. Self-reported data for this age group is important given the disagreement between adolescent- and parent-reported HRQL [6].

Previous studies on HRQL amongst adolescents with food allergy have focused on a wide range [4, 6] or unspecified [5] foods, as well as reported, rather than objectively diagnosed food allergies [5]. However, we believe that objectively diagnosed allergies to certain foods warrant particular attention. For example, allergies to the staple foods cow’s milk, hen’s egg [11] and wheat [12], typically present in infancy and often exist concomitantly [12]. Although these allergies often resolve by school age [11, 12], those with more severe symptoms or multiple food allergies may experience persistence of staple food allergy through later ages [11, 12]. As staple foods are ubiquitous in a typical western diet and are consequently difficult to avoid, the HRQL of adolescents experiencing disease persistence is likely to be impacted. Thus, we hypothesised that adolescents with staple food allergies would have poor HRQL, and that adolescents with a history of severe symptoms would have the worst HRQL. To this end, the aims of this study were to make use of the Swedish version of EuroPrevall’s FAQLQ-TF and to investigate the association between objectively-diagnosed staple food allergy and HRQL amongst adolescents.

## Methods

### Study design and participants

In this cross-sectional study, adolescents aged 13–17 years with paediatric allergist-diagnosed allergy to one or more staple foods (cow’s milk, hen’s egg and/or wheat) were identified from medical records and recruited in 2010–2012 by a paediatric nurse from the outpatient allergy clinic at Sachs’ Children and Youth Hospital, Södersjukhuset, in Stockholm, Sweden.

**Fig. 1:**
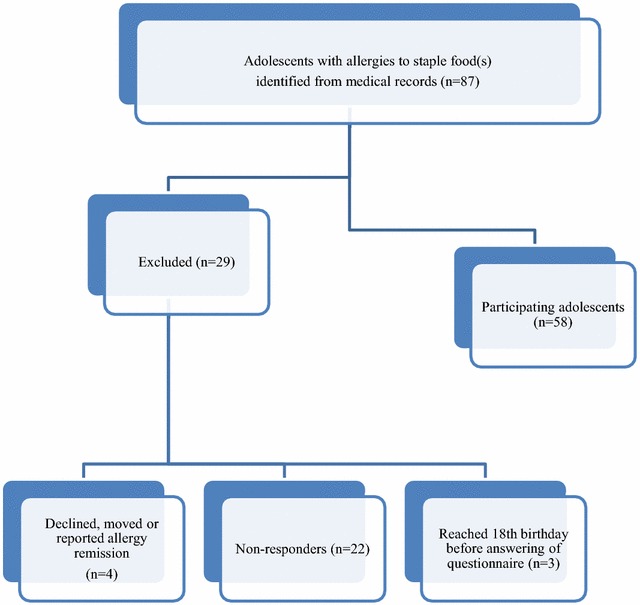
Flow chart detailing enrolment of adolescents with objectively-diagnosed staple food allergy

Inclusion criteria were a convincing history of allergy to one or more of the above-mentioned staple foods ascertained either by a positive food challenge with evident symptoms, or by levels of food specific Immunoglobulin E (IgE) antibodies levels associated with a 95 % probability of a positive result in a double-blind placebo controlled food challenge [13]. Exclusion criteria were an unclear allergy diagnosis to staple food(s), poor understanding of the Swedish language, or presence of coeliac disease, diabetes and/or a malignancy. Information on concomitant allergic disease (asthma, allergic rhinitis, allergic conjunctivitis, eczema) was also obtained. A total of 87 adolescents were eligible and invited to participate. These adolescents were mailed the FAQLQ-TF (described below, English version available as an Additional file 1), as well as an information letter and a postage-paid return envelope. Parents were mailed an information letter and consent form. Completed FAQLQ-TF and signed parental consent forms were received from 58 adolescents (67 % of those eligible; Fig. 1). Adolescents received two movie tickets following receipt of completed questionnaires. This study was approved by the Regional Ethical Review Board in Stockholm, Sweden (Dnr 2009/84-31/5). Personal data were treated according to the Swedish Personal Data Act.

### Exposures

Both the number of staple food allergies, as well as the number of offending foods (at least one staple food allergy and, participant-reported allergies to other foods) were considered as exposures.

Adolescents responded to 36 closed-ended questions on food allergy symptoms, from which we generated specific symptoms:

*Gastrointestinal:* stomach upset; vomiting; diarrhoea.

*Oral:* itchy tongue, mouth or lips; swollen tongue or lips.

*Upper respiratory:* runny or blocked nose; sneezing.

*Lower respiratory:* itchy or tight throat; difficulty swallowing; shortness of breath; wheeze; cough.

*Cardiovascular/neurological:* dizziness; tachycardia; blurred vision; inability to stand/collapse; loss of consciousness.

The most severe symptoms, including difficulty breathing, inability to stand/collapse, and/or loss of consciousness, involved the respiratory- and/or cardiovascular/neurological systems. In keeping with our previous publications on children [27] and adults [26], and approximating as best as possible the criteria outlined by Sampson et al. [14], such symptoms are collectively referred to as anaphylaxis. Adolescents were asked if they had been prescribed an adrenaline auto injector (AAI).

### Outcome

#### Food allergy quality of life questionnaire-teenager form (FAQLQ-TF)

The FAQLQ-TF [9] was translated into Swedish as per World Health Organization guidelines [15], and was piloted in 10 Swedish-speaking adolescents to ascertain comprehension. Following minor linguistic adjustments, the translation was deemed adequate. The FAQLQ-TF contains 28 questions on HRQL, each of which has corresponding closed-ended answers on a 7-point scale where 1 is best HRQL and 7 is worst HRQL [9]. Overall HRQL established by taking the mean of the 28 questions. These questions were also designed to address three domains: allergen avoidance and dietary restrictions (AADR), emotional impact (EI) and risk of accidental exposure (RAE). The first domain, AADR, describes adolescents’ perceptions of limitations, hesitations and refusals of foods that they purchase or are offered in social situations. The second domain, EI, reflects adolescents’ fears of an allergic reaction or accidental consumption of the food(s) to which they are allergic and their disappointment when others do not take their food allergy seriously. The third domain, RAE, captures adolescents’ assessments of needing to be cautious about purchasing food or eating out in relation to changes in ingredients, incorrect disclosure of ingredients and touching certain foods.

### Statistics

Floor and ceiling effects (percentages of patients with minimal and maximum scores, respectively) of the FAQLQ-TF were calculated to verify discriminative capacity. These effects were considered present if >15 % of a sample of a minimum of 50 individuals achieved the lowest or highest possible scores, respectively. Absence of these effects demonstrates the efficacy of the questionnaire.

Descriptive statistics included sample sizes (n), percentages, means, parametric two-sample t-tests and 95 % CI. Statistical significance was set at p < 0.05. Overall and domain-specific HRQL scores were calculated for the entire study population and stratified by gender. To permit statistical comparisons, the number of staple food allergies was classified into 2 dichotomous groups: 1 vs. 2–3. The number of offending foods was classified into 4 groups: 1, 2, 3 or >3. As described above, adolescents reported on symptoms. Adolescents may forget or inaccurately report their symptoms. Thus, we performed intra-class correlations of adolescent-reported symptoms with those reported by their parents as part of a parallel study [32] to measure reliability. These analyses showed modest correlations between adolescent- and parent-reported symptoms, with increasing reliability with increasingly severe symptoms (results not shown). As such, we present the results herein based on adolescent-reported symptoms.

Univariable and multivariable linear regression analyses were performed to identify predictors of HRQL. Potential covariates were identified based on prior knowledge of the exposures and outcome. The covariates gender, number of symptoms, history of anaphylaxis, AAI prescription and concomitant allergic disease were included in the final model as they statistically and independently altered the prediction model. The same models were used for overall and domain-specific HRQL. In keeping with previous publications on HRQL assessed via the FAQLQ, a score of ≥ ±0.5 was considered to be clinically relevant [9, 16]. Analysis was performed with STATA Statistical Software (release 13.1; StataCorp, College Station, Texas, USA).

## Results

The discriminant capacity of the FAQLQ-TF was confirmed as domain-specific floor and ceiling effects were below 15 % (results not shown).

Our study population included 58 adolescents, of whom 40 (69 %) were boys (Table 1). Most (62 %) of participants were allergic to only one staple food, although 7 % were allergic to all three staple foods. The most common staple food allergy was to hen’s egg (79 %). Other participant-reported allergies to other foods, particularly to tree nuts (60 %) and peanuts (53 %), were also common. Nearly all adolescents reported lower respiratory (95 %) and dermatological (90 %) symptoms. Gastrointestinal symptoms were also common (68 %). Although symptoms involving the cardiovascular/neurological system were the least common, they were nonetheless reported by 27 % of adolescents.

**Table 1:** Descriptive allergy characteristics of adolescents with objectively-diagnosed staple food allergy

	n	%
Sex		
Boys	40	69.0
Girls	18	31.0
Number of staple food allergies		
1	36	62.1
2	18	31.0
3	4	6.9
Offending staple foods^a^		
Hen’s egg	46	79.3
Cow’s milk	29	50.0
Wheat	5	8.6
Participant-reported allergies to other foods^a^		
Tree nuts	35	60.3
Peanuts	31	53.4
Fruit	17	29.3
Vegetables	8	13.8
Fish	9	15.5
Shellfish	12	20.7
Soy	6	10.3
Sesame seeds	2	3.4
Number of offending foods^b^		
1	8	13.8
2	10	17.2
3	13	22.4
>3	27	46.6
Symptoms resulting from staple foods^a^		
Gastrointestinal	28	68.3
Dermatological	37	90.2
Oral cavity	32	39.0
Upper respiratory	16	39.0
Lower respiratory	39	95.1
Cardiovascular	11	26.8
Anaphylaxis	23	56.1
Concomitant allergic disease^c^		
None or one	11	19.0
Two or more	47	81.0

^a^Not mutually exclusive

^b^Includes at least 1 objectively-diagnosed staple food, as well as any participant-reported allergies to other foods

^c^Asthma, allergic rhinitis, allergic conjunctivitis and/or eczema

**Fig. 2:**
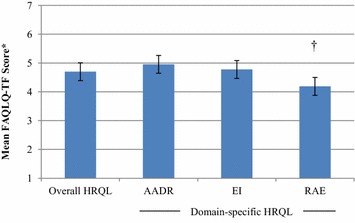
Overall- and domain-specific HRQL mean scores and 95 % CI for adolescents with objectively-diagnosed staple food allergy. *Asterisk* FAQLQ-TF on a scale of 1–7, where 1 corresponds to best HRQL and 7 corresponds to worst HRQL; based on self-report. † Compared to AADR (p < 0.001). *HRQL* health-related quality of life, *AADR* allergen avoidance and dietary restrictions, *EI* emotional impact, *RAE* risk of accidental exposure

Overall HRQL and domain-specific HRQL are presented in Fig. 2. The overall HRQL mean score was 4.70/7.00 (95 % CI 4.30–5.01). Girls had clinically worse, but not statistically significantly different HRQL than boys (5.12 ± 1.01 vs. 4.51 ± 1.23, respectively; mean difference = 0.71; p < 0.07).

With consideration to the different domains, the mean scores for AADR and EI were comparable (Fig. 2). In contrast, RAE was significantly associated with clinically better HRQL compared to the reference domain, AADR (mean 4.19/7.00; 95 % CI 3.82–4.56 vs. mean 4.95/7.00; 95 % CI 4.65–5.25, respectively; mean difference = 0.76; p < 0.001). Only the domain, EI, differed significantly between the sexes, with clinically worse HRQL amongst girls than boys (5.38 ± 1.4 vs. 4.50 ± 0.24; mean difference = 0.88; p < 0.04).

Investigation to the individual questions of each domain revealed further insights into the fine tuning of the HRQL of the different domains. For example, in the domain AADR, compared to the question with the highest mean score (i.e. worst HRQL), ‘How troublesome do you find it, because of your food allergy, that you must check yourself whether you can eat something when eating out’, factors that were associated with significantly better HRQL included limitations on eating, buying or refusing foods, or spontaneously accepting invitations to a meal. Within the domain, EI, compared to the reference question, ‘How disappointed are you when people don’t take your food allergy into account?,’ factors associated with significantly better HRQL included feeling discouraged, carrying an AAI, or fears related to eating something ‘wrong’ or something new. Within the domain, RAE, compared to the reference question, ‘How troublesome do you find it, because of your food allergy, that you have to explain to people around you that you have a food allergy?,’ the only factor associated with significantly better HRQL related to labelling discrepancies between bulk and individual packaging (Table 2).

**Table 2:** Mean scores for individual questions used to calculate domain-specific HRQL

	Mean	*p* value^†^
Allergen avoidance and dietary restrictions		
How troublesome do you find it, because of your food allergy, that you:		
Must check yourself whether you can eat something when eating out?	5.76	
Must read labels?	5.74	0.95
Hesitate eating a product when you have doubts about it?	5.67	0.75
Must always be alert to what you are eating?	5.66	0.70
Are less able to taste or try various products when eating out?	5.36	0.15
Are able to eat fewer products?	5.21	<0.05
Are limited as to the products that you can buy?	4.47	<0.001
Are less able to spontaneously accept an invitation to stay for a meal?	4.09	<0.001
Must be careful about touching certain foods?	3.47	<0.001
Must refuse treats at school or work?	2.58	<0.001
Emotional impact		
Answer the following:		
How disappointed are you when people don’t take your food allergy into account?	5.50	
How discouraged do you feel during an allergic reaction?	4.74	<0.05
How troublesome do you find it, because of your food allergy, that you:		
Have the feeling that you have less control of what you eat when eating out?	5.40	0.86
Must carry an EpiPen®?	4.36	<0.01
How frightened are you because of your food allergy:		
Of accidentally eating something wrong?	4.59	<0.05
Of an allergic reaction?	4.53	<0.05
To eat something that you have never eaten before?	4.31	<0.05
Risk of accidental exposure		
How troublesome do you find it, because of your food allergy, that you:		
That you have to explain to people around you that you have a food allergy?	5.00	
That during social activities others can eat the food to which you are allergic?	4.95	0.88
That during social activities your food allergy is not taken into account enough?	4.47	0.17
That the ingredients of a food change?	4.45	0.10
That the label states: “May contain (traces of)….”?	4.43	0.15
That the labelling of the bulk packaging (e.g. box or bag) is different than the individual packages?	3.27	<0.001

FAQLQ-TF on a scale of 1–7, where 1 corresponds to best HRQL and 7 corresponds to worst HRQL; based on self-report

^†^Compared to the individual question with the highest mean score (i.e. worst HRQL) within each domain

In linear regression analyses adjusted for sex, number of symptoms, history of anaphylaxis, AAI prescription and concomitant allergic disease (excluding the predictor), girls had clinically worse and but not statistically significant HRQL than boys (*B* = −0.58; 95 % CI −1.34; 0.19; p = 0.13; Table 3). Similarly, allergies to >3 staple food allergies and AAI prescription reached the threshold of ≥0.5 for clinical relevance but only trended towards significance (p = 0.13 and p = 0.06, respectively).

**Table 3:** Linear regression analyses of HRQL for adolescents with objectively-diagnosed staple food allergy

	n	*B*	95 % CI for *B*	p value
Sex				
Boys	18	Ref		
Girls	40	−0.58	−1.34; 0.19	0.13
Number of staple food allergies				
1	36	Ref		
2–3	22	0.37	−0.31; 1.06	0.28
Number of offending foods^a^				
1	10	Ref		
2	9	0.79	−0.43; 2.01	0.20
3	13	0.03	−1.11; 1.17	0.95
>3	26	0.82	−0.24; 1.89	0.13
Number of symptoms resulting from staple foods				
0–3	15	Ref		
4–6	26	0.10	−0.87; 1.07	0.83
Adrenaline auto injector possession				
No	14	Ref		
Yes	44	0.78	−0.02; 1.57	0.06

Adjusted for the covariates: sex, number of symptoms, history of anaphylaxis, adrenaline auto injector prescription and concomitant allergic disease, excluding predictor

^a^ Includes at least 1 objectively-diagnosed staple food, as well as participant-reported allergies to other foods

Consideration to the presence vs. absence of specific symptoms including anaphylaxis attributable to staple foods yielded no statistically significant associations. Only previous lower respiratory symptoms reached the threshold for clinically better HRQL (*B* = 0.67; 95 % CI −1.37; 2.70, p = 0.51; Table 4).

**Table 4:** Linear regression analyses for presence vs. absence of specific symptoms resulting from staple foods in adolescents with objectively-diagnosed staple food allergy

	n	*B*	95 % CI for *B*	p value
Gastrointestinal				
No	13	Ref		
Yes	28	0.02	−1.04; 1.08	0.97
Dermatological^a^				
No	4	Ref		
Yes	37	0.41	−1.86; 2.68	0.72
Oral				
No	9	Ref		
Yes	32	0.41	−0.98; 1.80	0.57
Upper respiratory				
No	25	Ref		
Yes	16	0.29	−0.88; 1.44	0.62
Lower respiratory^a^				
No	2	Ref		
Yes	39	0.67	−1.37; 2.70	0.51
Cardiovascular				
No	30	Ref		
Yes	11	0.29	−0.88; 1.45	0.62
History of anaphylaxis				
No	18	Ref		
Yes	23	−0.33	−1.23; 0.58	0.46

Adjusted for the covariates: sex, number of symptoms, history of anaphylaxis, adrenaline auto injector prescription and concomitant allergic disease, excluding predictor

Non-mutually exclusive symptoms

^a^ Interpret with caution due to small counts

## Discussion

In this cross-sectional study of adolescents with objectively-diagnosed staple food allergies to cow’s milk, hen’s egg and/or wheat, overall HRQL, as ascertained by a food allergy specific questionnaire, was poorer than average. The domain risk of accidental exposure was significantly associated with clinically better HRQL than the domain allergen avoidance and dietary restrictions. Girls had clinically worse, but not statistically significantly different HRQL than boys. HRQL tended to be worse amongst adolescents with allergies to more than three foods or those who had been prescribed an AAI. In contrast, the number and type of previous symptoms and history of anaphylaxis were not associated with worse HRQL.

We highlight the key strengths of this study we used a food allergy-specific questionnaire that is robust for adolescents with food allergies [9, 17] that provided insights into associations between food allergy and HRQL that could not have been gleaned via a generic questionnaire [9]. Similarly, this work presents the first results of the Swedish version of the FAQLQ-TF. In our study, participants had objectively-diagnosed allergies, thus providing insight into the impact of true, rather than perceived, food allergy on HRQL. Amongst children, HRQL does not differ between these two phenotypes [18]. However, consideration of the association between objectively-diagnosed allergy and adolescent-reported HRQL warrants consideration as this age group is increasingly responsible for their own food choices, and thus the potential consequences of inappropriate food choices. Although adolescents’ food choices are influenced by their allergies, they also make choices based on peers and sensory preferences [19]. We believe that we are the first group to report on HRQL amongst adolescents with allergies to foods that are ubiquitous in a typical western diet, but which also receive less attention in relation to HRQL than other common food allergens, such as peanuts or tree nuts.

We also acknowledge the limitations of our study. Our study was cross-sectional in design, thereby precluding establishment of a causal relationship between staple food allergy and HRQL. As well, our study population included more boys than girls. As evidenced in the general adolescent population [20], and amongst food hypersensitive adolescents for whom HRQL was established using a generic questionnaire [5], boys generally have better HRQL than girls. Thus, we surmise that, had adolescent boys and girls been equally represented, overall HRQL may have been even worse.

The FAQLQ-TF is specifically designed to capture adolescents’ perceptions of HRQL [9]. Elsewhere, responses from Dutch adolescent-parent pairs to the FAQLQ have been compared [6]. In that study, adolescents were allergic to a wide range of common food allergens, but staple food allergy did not predominate. Amongst these adolescent-parent pairs, adolescents reported clinically worse, but not statistically significant differences in HRQL. This finding underscores the need to specifically query adolescents’ perceptions of HRQL and address not only statistical differences but consideration to the magnitude of the differences. Interestingly, our domain-specific scores exceeded the clinically relevant difference of ≥0.5 compared to the Dutch study [6], as well as other studies in which the FAQLQ-TF was used [6, 9, 17, 21]. Collectively, these studies suggest that staple food allergy is associated with worse HRQL than allergies to other foods. One can speculate that this may be due to the ubiquity of staple foods in a typical western diet, making them challenging to avoid. This challenge may be compounded by the fact that, although many countries have regulatory frameworks for allergens contained in processed foods [22], such labelling most commonly identifies non-staple foods [23] and may contribute to confusion and complacency amongst food allergic individuals [24, 25].

We identified that staple food allergy impacted on adolescents’ lifestyles, as underscored by poorer than average overall HRQL mean score [i.e. better HRQL], as well as within the domain, AADR. Others have reported similar findings on the impact of food allergy on adolescents’ lifestyles [6]. The similarities between these findings are not surprising, as adolescents spend increasing amounts of time in social settings away from home. However, our findings extend those of previous studies, as we were able to disentangle the specific factors that contributed most to worse HRQL.

In the present study, adolescents reported worse HRQL related to emerging independence and the need for support, as evidenced by domain-specific mean scores which were highest for the questions relating to needing to check for themselves if they can eat a food whilst dining out, expressing disappointment when their food allergies are not taken into account, or explaining to others about their food allergy. Previously, we reported that differences between the domains were present amongst adults [26], but not children [27] with staple food allergy. As with adults [26] and children [27], adolescents with staple food allergy reported significantly worse HRQL if they had multiple food allergies or possessed an AAI. Likewise, both adolescents and adults [26] reported worse HRQL within the domain AADR. As different domains were defined for children, a comparison of the domains between children and adolescents is not possible. In contrast to both adults [26] or children [27], adolescents did not report worse HRQL in association with a history of anaphylaxis or the number and type of symptoms. Taken together, it could be speculated that children may not feel burdened by the dietary restrictions imposed by staple food allergy, likely as the responsibility for safe food choices is assumed by their parents/guardians, whereas adolescents and adults do have to assume this responsibility. The burden of dietary restrictions and safe food choices has also been qualitatively explored amongst adolescents. Unlike non-food allergic adolescents, those with food allergies feel safe under parental control and thus do not necessarily want to make food-related decisions independently [19]. Adolescents with food allergy describe themselves as being very mature for their age, yet dependent on others in the event of a reaction [28]. One can speculate that these opposing characteristics may result in worse food allergy-specific HRQL.

Unlike other chronic conditions which adolescents may neglect or be non-compliant [29], food allergy cannot be ignored for longer than an interval between meals or snacks. But given that food is an integral part of social events, feelings of exclusion and ‘being different’ may ensue [7]. This may begin to explain why food allergy is strongly associated with worse HRQL. This may also explain why neither the number of allergies or symptoms, nor AAI prescription is a predictor of clinically worse HRQL. Further, adolescents often base their food choices primarily on enjoyment and secondarily on content [19], thereby engaging in risk taking behaviours [30] that may potentially lead to severe reactions. Yet, like others [9], we found no difference in HRQL between those with vs. without a history of anaphylaxis.

The financial burden of food allergy on healthcare systems [31] and on households [32] is high, and allergy-related hospitalisations are increasing [33]. Worse HRQL also predicts greater healthcare costs [34]. Thus, addressing HRQL amongst adolescents, as well as children [27] and adults [26] with staple food allergy warrants considerable attention.

## Conclusions

As ascertained via a food allergy-specific questionnaire, adolescents with staple food allergy report poorer than average HRQL, specifically in relation to emerging independence and the need for support. Girls have clinically worse HRQL than boys. The number and type of previous symptoms and history of anaphylaxis were not associated with worse HRQL (Additional file 1.).

## Additional file

10.1186/s13601-016-0128-5 Food allergy quality of life questionnaire–teenager form (13–17 years).

## Abbreviations

AADR: allergen avoidance and dietary restrictions
AAI: adrenaline auto-injector
EI: emotional impact
FAQLQ-TF: food allergy quality of life questionnaire-teenager form
HRQL: health-related quality of life
RAE: risk of accidental exposure
95 % CI: 95th percent confidence interval
