# Supplementary material for: Impaired health-related quality of life in adolescents with allergy to staple foods
Source: Clin Transl Allergy. 2016 Sep 30;6:37. doi: 10.1186/s13601-016-0128-5 (PMC5045620; doi:10.1186/s13601-016-0128-5)
Supplement: Supplementary file 1 — 10.1186/s13601-016-0128-5 Food allergy quality of life questionnaire–teenager form (13–17 years). [file 13601_2016_128_MOESM1_ESM.pdf]

English VS– Teenager version

EuroPrevall – ID

Centre-No. WP-No. Patient Number

FAQLQ-TF

Food Allergy Quality of Life Questionnaire – Teenager Form (13-17 years)

You have been given this questionnaire by your clinician as part of your participation in the EuroPrevall project. This questionnaire should be returned to researchers at the University Medical Center Groningen (UMCG). Researchers at the UMCG do not have access to your personal details (your name, address). All the information you provide will be encrypted with a code number; only your clinician is able to link this number with your personal details. The information you provide in the questionnaire will only be seen by researchers at the UMCG and will be analysed scientifically and anonymously. The findings will be published along with the information provided by all other participants in this survey. All the information you provide will be strictly CONFIDENTIAL and completely anonymous.

Part A

The following questions concern the influence your food allergy has on your quality of life. Answer every question by marking the appropriate box with an 'x'. You may choose from one of the following answers.

0	1	2	3	4	5	6
not	barely	slightly	moderately	quite	very	extremely

	**How *troublesome* do you find it, because of your food allergy, that you …**	**0**	**1**	**2**	**3**	**4**	**5**	**6**
1	must always be alert as to what you are eating?	☐	☐	☐	☐	☐	☐	☐
2	are able to eat fewer products?	☐	☐	☐	☐	☐	☐	☐
3	are limited as to the products you can buy?	☐	☐	☐	☐	☐	☐	☐
4	must read labels?	☐	☐	☐	☐	☐	☐	☐
5	have the feeling that you have less control of what you eat when eating out?	☐	☐	☐	☐	☐	☐	☐
6	are less able to spontaneously accept an invitation to stay for a meal?	☐	☐	☐	☐	☐	☐	☐
7	are less able to taste or try various products when eating out?	☐	☐	☐	☐	☐	☐	☐
8	must check yourself whether you can eat something when eating out?	☐	☐	☐	☐	☐	☐	☐
9	hesitate eating a product when you have doubts about it?	☐	☐	☐	☐	☐	☐	☐
10	must refuse treats at school or work?	☐	☐	☐	☐	☐	☐	☐
11	must be careful about touching certain foods?	☐	☐	☐	☐	☐	☐	☐
12	must carry an Epipen? (If you don't have a Epipen mark an 'x' here ☐)	☐	☐	☐	☐	☐	☐	☐

0	1	2	3	4	5	6
not	barely	slightly	moderately	quite	very	extremely

**How *troublesome* is it, because of your food allergy, …**	**0**	**1**	**2**	**3**	**4**	**5**	**6**
13 that the ingredients of a food change?	☐	☐	☐	☐	☐	☐	☐
14 that the label states: “May contain (traces of)….”?	☐	☐	☐	☐	☐	☐	☐
15 that the labeling of the bulk packaging (for example box or bag) is different than the individual packages?	☐	☐	☐	☐	☐	☐	☐
16 that you have to explain to people around you that you have a food allergy?	☐	☐	☐	☐	☐	☐	☐
17 that during social activities others can eat the food to which you are allergic?	☐	☐	☐	☐	☐	☐	☐
18 that during social activities your food allergy is not taken into account enough?	☐	☐	☐	☐	☐	☐	☐

**How *frightened* are you because of your food allergy …**	**0**	**1**	**2**	**3**	**4**	**5**	**6**
19 of an allergic reaction?	☐	☐	☐	☐	☐	☐	☐
20 of accidentally eating something wrong?	☐	☐	☐	☐	☐	☐	☐
21 to eat something you have never eaten before?	☐	☐	☐	☐	☐	☐	☐

**Answer the following questions:**	**0**	**1**	**2**	**3**	**4**	**5**	**6**
22 How *discouraged* do you feel during an allergic reaction?	☐	☐	☐	☐	☐	☐	☐
23 How *disappointed* are you when people don’t take your food allergy into account?	☐	☐	☐	☐	☐	☐	☐

Part B

The following four questions are about the chance that you think you have of something happening to you because of your food allergy. Choose one of the answers. This is followed by two more questions about your food allergy. Answer every question by putting an 'x' in the box next to the proper answer.

0	1	2	3	4	5	6
**never (0% chance)**	**very small chance**	**small chance**	**fair chance**	**great chance**	**very great chance**	**always (100% chance)**

	**How great do you think the chance is that you ...**	**0**	**1**	**2**	**3**	**4**	**5**	**6**
**1**	will accidentally eat something to which you are allergic?	☐	☐	☐	☐	☐	☐	☐
**2**	will have a severe reaction if you accidentally eat something to which you are allergic?	☐	☐	☐	☐	☐	☐	☐
**3**	will die if you accidentally eat something to which you are allergic?	☐	☐	☐	☐	☐	☐	☐
**4**	can **not** effectively deal with an allergic reaction should you accidentally eat something to which you are allergic?	☐	☐	☐	☐	☐	☐	☐

**5. How many products must you avoid because of your food allergy?**	**6. How great is the impact of your food allergy on your social life?**
☐ almost none	☐ negligibly small
☐ very few	☐ very small
☐ a few	☐ small
☐ some	☐ moderate
☐ many	☐ great
☐ very many	☐ very great
☐ almost all	☐ extremely great

Part C

Finally, a number of general questions concerning your food allergy are listed below. Place an 'x' next to the answers which apply to you.

1. Today's date …. - …. - …….. (dd-mm-yyyy)
2. Date of birth …. - …. - …….. (dd-mm-yyyy)
3. I am a ☐ boy ☐ girl

4. To which food(s) are you allergic? *(Check all that apply!)*

☐ Peanut ☐ Egg ☐ Sesame seed ☐ Celery
☐ Nuts ☐ Wheat ☐ Fish ☐ Fruits
☐ Milk ☐ Soy ☐ Shell fish ☐ Vegetables
☐ Other, namely……………...………………………………………………………………………..

5. What happened to you during the worst food allergy attack you have ever had?
(Check all that apply!)

☐ itchy mouth
☐ itchy throat
☐ itchy ears
☐ itchy tongue
☐ itchy lips
☐ swollen tongue
☐ swollen lips
☐ runny nose
☐ blocked nose
☐ sneezing
☐ itchy eyes
☐ watery eyes
☐ red eyes
☐ tightening throat
☐ difficulty swallowing
☐ hoarseness / hoarse voice
☐ difficulty breathing
☐ shortness of breath
☐ wheezing
☐ cough
☐ itchy skin
☐ red rash
☐ swelling of the skin
☐ hives
☐ worsening eczema
☐ sick to your stomach
☐ stomach cramps
☐ vomiting
☐ diarrhea
☐ dizziness
☐ feeling your heart beat fast
☐ loss of vision
☐ inability to stand
☐ light headedness
☐ collapse
☐ loss of consciousness / passing out

6. Which food was the cause of the worst food allergy attack described above?
……………………………………………………………………………………………………….

7. How long ago did the worst food allergy attack described above occur?
……………………………………………………………………………………………………

8. Who diagnosed your food allergy? *(Check all that apply!)*

☐ I, myself ☐ allergist ☐ alternative practitioner
☐ family doctor / GP ☐ dermatologist ☐ other, namely ………………..
☐ dietician ☐ pediatrician …………………………………….

9. Were you prescribed an epinephrine / adrenaline auto injector? ☐ yes ☐ no
(e.g. EpiPen, Twinject, Anapen)

This is the end of the questionnaire.

Thank you for your participation!
